# Sweet Syndrome due to Myelodysplastic Syndrome: Possible Therapeutic Role of Intravenous Immunoglobulin in Addition to Standard Treatment

**DOI:** 10.1155/2010/328316

**Published:** 2010-03-29

**Authors:** Harry H. S. Gill, Anskar Y. H. Leung, N. J. Trendell-Smith, C. K. Yeung, Raymond Liang

**Affiliations:** ^1^Division of Haematology, Medical Oncology and Bone Marrow Transplantation, Department of Medicine, Queen Mary Hospital, The University of Hong Kong, Pokfulam, Hong Kong; ^2^Department of Pathology, Queen Mary Hospital, Pokfulam, Hong Kong; ^3^Division of Dermatology, Department of Medicine, The University of Hong Kong, Queen Mary Hospital, Pokfulam, Hong Kong

## Abstract

We report an 82-year-old lady who developed sudden onset nodular and erythematous lesions and neutrophilia following an episode of urinary tract infection. Skin biopsy confirmed the diagnosis of Sweet syndrome. Response to the use of prednisolone alone was not satisfactory. The skin lesions however showed a sustained response to the regular use of intravenous immunoglobulin (IVIG) and prednisolone was slowly weaned off. Our case highlights the possible therapeutic role of IVIG in managing this condition.

## 1. Introduction

 Sweet syndrome, also termed acute febrile neutrophilic dermatosis, is the prototype of neutrophilic dermatoses. Histologically, Sweet syndrome is characterized by a diffuse infiltrate of mature neutrophils in the dermis without vasculitis. Eosinophils, lymphocytes, and histiocytes may be observed in variable numbers and a histiocyte-rich stage in the evolution of Sweet syndrome has been described. Differential diagnoses include infection, pyoderma gangrenosum, other neutrophilic dermatoses and vasculitides. The use of systemic corticosteroids is the mainstay in the management of Sweet syndrome. Alternative agents, for instance, dapsone and colchicines, are used in cases of relapse or refractoriness to corticosteroids.

 We describe the clinical course of an 82-year-old lady with myelodysplasia who developed rapidly progressive neutrophilia and pustulovesicular skin lesions following an episode of febrile illness. Skin biopsy confirmed the diagnosis of Sweet syndrome. The response to systemic corticosteroids was modest with relapse during treatment. The skin lesions and neutrophilia showed a prompt and durable response to the regular use of intravenous immunoglobulin. This provides evidence to the possible therapeutic role of IVIG in Sweet syndrome in addition to the use of corticosteroids and other anti-inflammatory agents.

## 2. Case Report

**Figure 1:**
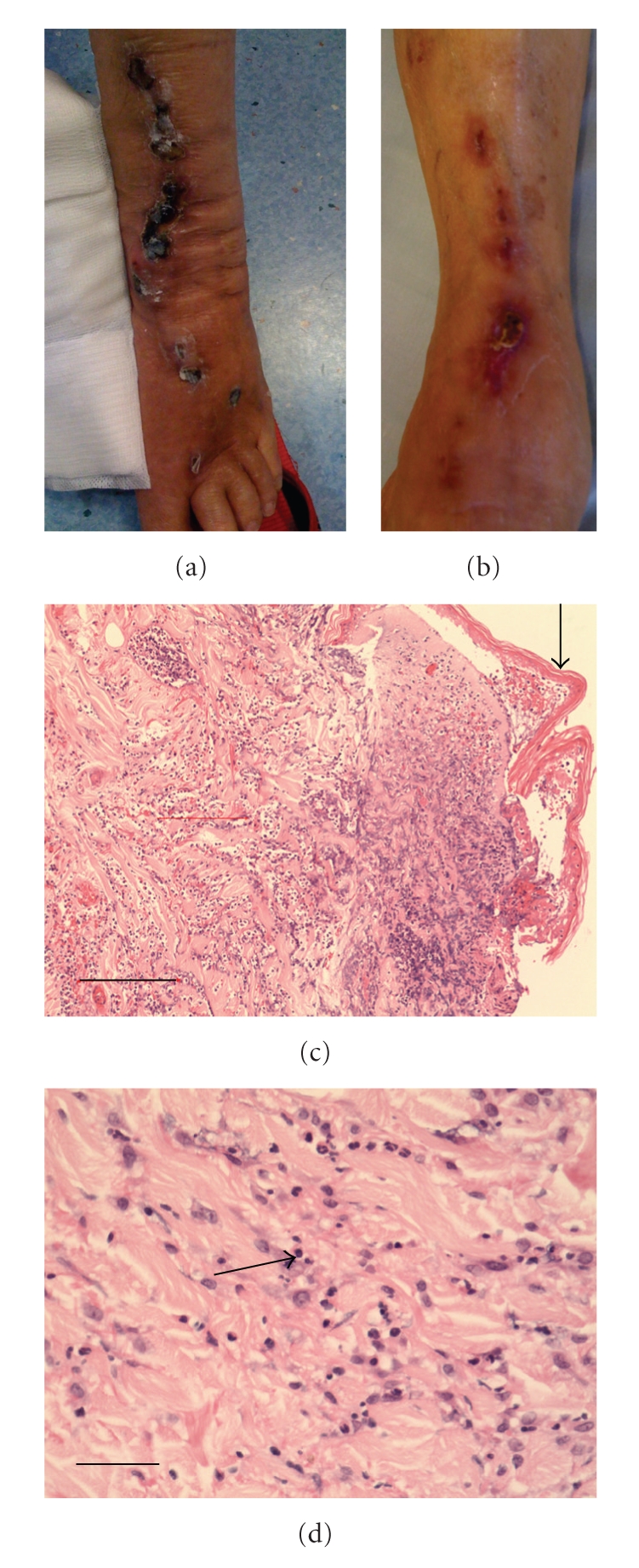
Clinicopathologic features of Sweet syndrome in our patient at diagnosis: (a) Photograph showing the purpuric patches and pustulovesicular lesions over the left leg and dorsum of foot at diagnosis. (b) Photograph showing the resolving skin lesions over the left leg and dorsum of foot at the time of writing the manuscript. (c) Low power H & E of skin showing diffuse infiltrate of dermis and superficial subcutis with neutrophils (arrow). There is incipient necrosis of the epidermis. Dimension bar (left lower corner) 250 microns. (d) High power H & E of skin—the arrow shows focal leukocytoclasis without vasculitis—dimension bar (left lower corner) 50 microns.

 An 82-year-old lady with 10-year history of diabetes mellitus, hypertension, and sick sinus syndrome presented in February 2008 with anaemia and thrombocytopenia. Bone marrow aspiration and biopsy confirmed myelodysplastic syndrome (myelodysplastic/myeloproliferative neoplasm, unclassifiable under the WHO criteria 2008) with normal cytogenetics. She was treated conservatively with regular blood transfusion. In November 2008, she developed fever, confusion, and marked renal impairment with a creatinine of 350 *μ*mol/L. Her white cell count (WCC) was 57.47  × 10^9^/L (Neutrophils: 43.18×10^9^/L, Lymphocytes 1.58  × 10^9^/L, Monocytes 1.67  × 10^9^/L, Eosinophil 0.28 × 10^9^/L, Basophils 1.07 ×  10^9^/L). Frequent myeloid precursors and band forms were present. There were no circulating blasts. Urine culture grew *E. coli* and *Klebsiella* species sensitive to standard antimicrobials. Blood cultures at presentation were negative for bacteria or fungi. In light of her immunocompromised state and clinical sepsis, she was treated empirically with meropenem. Despite treatment with meropenem, her neutrophil counts continued to rise and her fever only showed a modest response. Repeated sepsis workup including urine and blood cultures was negative for any microorganisms. On the next day of admission, pustulovesicular eruptions developed over her arms and purpuric patches over the left thigh, shin, and dorsum of her left foot (Figure 1(a)). A skin biopsy of a suppurative lesion on her left thigh showed a diffuse neutrophilic infiltrate within the mid to upper dermis, associated with early leukocytoclasis and absence of vasculitic changes (Figures 1(c) and 1(d)). There was no evidence of leukaemic infiltration and microbiological investigations showed no infective organisms. The diagnosis of Sweet syndrome (acute febrile neutrophilic dermatosis) was made. Her skin condition deteriorated with exudation and new lesions over the skin biopsy site. In addition, there was evidence of epidermal necrosis over the left ankle. Bone marrow examination did not show any evidence of leukaemic transformation.

**Figure 2:**
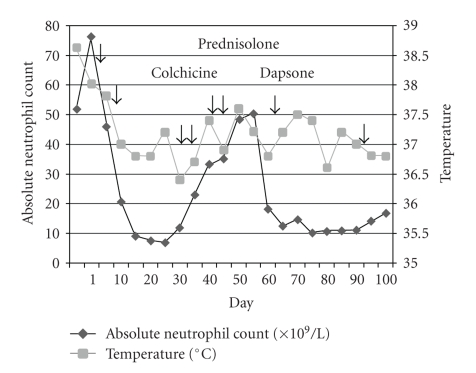
The effect of various pharmacotherapies on the absolute neutrophil count and temperature (each arrow represents a 21-gram-dose of IVIG).

 Given her immunocompromised state secondary to MDS, she was given a single dose of intravenous immunoglobulin (IVIG) at 21 grams (0.5 g/kg) 5 days after admission with the intention of promoting recovery from infection and due to the possibility of viral infections in light of fever and rash. Intriguingly, both the fever and the neutrophilia responded promptly (Figure 2). Thereafter prednisolone (30 mg daily) was started as standard treatment for Sweet syndrome. In view of the initial response to IVIG, another dose of 21 grams was given 5 days after the initial dose. The pustulovesicular lesions and her renal function also showed progressive improvement. 

 Her white cell count gradually dropped to 10.4 × 10^9^/L (absolute neutrophil count 9.2 × 10^9^/L) 3 weeks after her admission. Prednisolone was gradually tailed down within three weeks to 5 mg daily. However, her skin condition deteriorated with worsening ulceration over the left thigh and new nodular lesions on the left arm and leg. At the same time, her neutrophil count also increased. Prednisolone was stepped up to 40 mg per day. In addition to that, two consecutive doses of IVIG were given. Colchicine was added but was discontinued after four days due to diarrhea. Her skin condition was stabilized but the neutrophil count continued to rise. IVIG was administered at the same dose for two consecutive days a week later. Dapsone was added on day 55 of her admission and was given for 10 days. The neutrophil count dropped thereafter. Her renal function normalized 2 months after initial presentation. Monthly dose of IVIG was administered and prednisolone could be slowly tailed off without worsening of her skin condition at the time of writing this paper 8 months after presentation (Figure 1(b)).

## 3. Discussion

 Clinically, Sweet syndrome is characterized by systemic upset including fever and malaise, and peripheral neutrophilia. The cutaneous features include painful and nonpruritic asymmetrical erythematous plaques, papules, and nodules of around 2 to 3 centimeters mostly affecting the upper extremities, face, and neck [1, 2]. Pathergy phenomenon has been described in which skin lesions occurred at biopsy, venepuncture, and intravenous drip sites. Involvement of eyes as well as transient renal, liver, pancreatic, neurologic and psychiatric manifestations has been described but is unusual [3]. Sweet syndrome has a marked female preponderance and it affects primarily the middle age population [3, 4]. Most cases were associated with underlying malignancy, notably myelodysplastic syndrome, acute myeloid leukaemia, and myeloproliferative disorders and less commonly breast cancer and solid organ tumors of the genitourinary and gastrointestinal tracts. 

The pathogenesis of Sweet syndrome is poorly understood. Immune complex vasculitis, T-cell activation, and altered neutrophil function have been postulated but experimental evidence was lacking. More recently, deregulated secretion of interleukins 1, 3, 6, and 8, granulocyte colony-stimulating factor (G-CSF), granulocyte-macrophage colony-stimulating factor (GM-CSF), and interferon gamma have been reported [5].

 The optimal treatment for Sweet Syndrome has not been defined. Prednisolone is the mainstay of treatment. Rapid response is often seen but recurrence is frequently seen. Steroid refractory and/or relapsed diseases are often treated with nonsteroidal anti-inflammatory drugs, colchicine, dapsone, potassium iodide, doxycycline and clofazimine [1, 3], and more recently etanercept, a tumor necrosis factor alpha (TNF-*α*) antagonist [6], based on the hypothesis that TNF-*α* activates neutrophils in this disease.

 Our patient presented with systemic upset, fever, neutrophilia and typical cutaneous, and histological features of Sweet syndrome. Surprisingly, IVIG resulted in a prompt and dramatic response in fever and neutrophilia. Such response was clearly observed before the commencement of prednisolone. In addition, the skin condition showed a progressive and sustained improvement with regular administration of IVIG. The exact mechanism is not known. However, our observations should be interpreted with caution since prednisolone, colchicines, and dapsone have also been used and it was likely that they might have contributed to the improvement of the patient's condition. Whether these agents, when used with IVIG, might provide synergism to the latter would also have to be carefully examined. Secondly, the initial response of fever and neutrophilia to IVIG might be related to its passive immunity in addition to the use of broad spectrum antibiotics. Notwithstanding this limitation, our clinical observation might provide us with a ground for further evaluating the clinical efficacy of IVIG in the treatment of this disease. Recently, Haliasos et al. [7] also reported a 4-year-old girl with chronic recurrent Sweet syndrome associated with primary immunodeficiency and thrombocytopenia being successfully treated with IVIG and dapsone. Therefore, our case report provided the first piece of evidence that IVIG ameliorated the clinical course of Sweet syndrome independent of the immunodeficiency state.
